# Adult presentation of severe sequelae of Tom Smith arthritis treated with bilateral total hip arthroplasty: A case report

**DOI:** 10.1016/j.ijscr.2022.107090

**Published:** 2022-04-18

**Authors:** Umesh Jayarajah, Rukshan Sooriyarachchi

**Affiliations:** Department of Orthopaedics and Trauma, National Hospital of Sri Lanka, Colombo, Sri Lanka

**Keywords:** Tom Smith arthritis, Pyogenic arthritis, Total hip arthroplasty, Hip dysplasia, Case report

## Abstract

**Introduction and importance:**

Tom Smith arthritis (TSA) is a pyogenic arthritis of the hip joint that occurs in infancy and has considerable morbidity. Reports on surgical management of severe hip dysplasia in adulthood secondary to TSA are extremely limited. We describe a patient who successfully underwent a bilateral total hip arthroplasty for the severely damaged hip joints secondary to TSA with satisfactory functional outcomes.

**Case presentation:**

A 25-year-old female was unable to walk for more than 10 ft due to pain in both hips and knees predominantly on the left side. She developed pyogenic septic arthritis with sepsis at 6 weeks of age and underwent multiple surgical procedures to drain the infection and for reconstruction. She had limited range of motion and was severely disabled. She underwent an uneventful left total hip arthroplasty and two years later, a right total hip arthroplasty using S-ROM modular hip systems. The pre and post-operative Harris Hips scores were 53.4 (left), 46 (right) and 95.7 (left), 89.65 (right), respectively.

**Clinical discussion:**

Detailed preoperative evaluation of the anatomy was paramount. Assessment of the limb-length discrepancy by means of scanogram, templating the anatomy with computed tomography and planning the anatomical location of the centre of the relocated hip were mandatory.

**Conclusion:**

Bilateral total hip arthroplasty is a feasible option to manage the rare occurrence of severely damaged bilateral hip joints caused by TSA presenting in adulthood. Reconstructive options for late sequelae should be individualized based on the degree of involvement, hip stability, and patient expectations.

## Highlights

• Reconstructive options for late sequelae of Tom Smith arthritis should be individualized.
• Pre-operative assessment with scanogram and computed tomography is paramount.
• Bilateral total hip arthroplasty is a feasible option for severe late sequalae of Tom Smith arthritis.

## 1 Introduction

Tom Smith arthritis is a pyogenic arthritis of the hip joint that occurs in infancy caused by nontuberculous bacteria. The femoral head at this age is completely cartilaginous and vulnerable to destruction due to infection. Therefore, Tom Smith arthritis is associated with considerable morbidity and mortality affecting the long-term quality of life [1]. The prognosis of infantile septic arthritis is related to the age, delay in treatment and the causative organism [2]. Furthermore, due to the deep location, difficulty in diagnosis and difficulties in aspiration and washouts, hip joint arthritis is associated with poor outcomes [3]. Septic arthritis in neonates and infants are associated with poorer outcomes compared to that occur in children [4].

Tom Smith arthritis can lead to considerable long-term morbidity such as acetabular dysplasia, subluxation and dislocation, cartilage necrosis, premature or abnormal closure of triradiate cartilage, limb-length discrepancy, abnormal or premature closure of the proximal femoral physis, ischemic necrosis and complete destruction of the femoral head and femoral neck pseudoarthrosis [4]. Several studies have described the surgical management of such complications in children and adolescents [5], [6], [7]. However, literature on surgical management of severe hip sequelae in adulthood secondary to infantile septic arthritis is extremely limited. Such management is challenging and the outcomes are unpredictable due to the lack of approved guidelines and consensus. In the following report, we describe a patient who successfully underwent a bilateral total hip arthroplasty for the severely damaged hip joints secondary to Tom Smith arthritis. The work has been reported based on the SCARE 2020 criteria [8].

## 2 Case presentation

A 25-year-old female self-presented to the orthopedic consultation due to inability to walk for more than 10 ft due to pain in both hips and knees. At presentation, the pain was worse on the left side. Her hip abduction was limited bilaterally and the knees were rubbing each other while walking. She also complained of inability to sit for more than an hour due to pain in both hips. She was unemployed and unmarried. There was no distal neurovascular compromise. She had no significant drug or allergy history, family history or psychosocial history. Her body mass index was 24.8 kg/m^2^.

Her past medical history includes septicaemia with septic arthritis of both hips (Tom Smith arthritis) with multiple abscesses and osteomyelitis of femoral metaphyses at the age of 6 weeks. She underwent bilateral arthrotomies and wash out followed by Gallow's traction for 17 days. Furthermore, she underwent an open reduction of the left hip joint at 4 years for correction of abnormal lateral rotation and reconstruction of the destroyed femoral head using a fibular bone graft. Furthermore, reconstruction of right hip was performed at 6 years using a 3 in. segment of ulna bone to reconstruct the femoral head. The surgeries were uneventful. However, she continued to have bilateral hip and knee pain and difficulty in walking which were progressive and severely disabling.

**Fig. 1:**
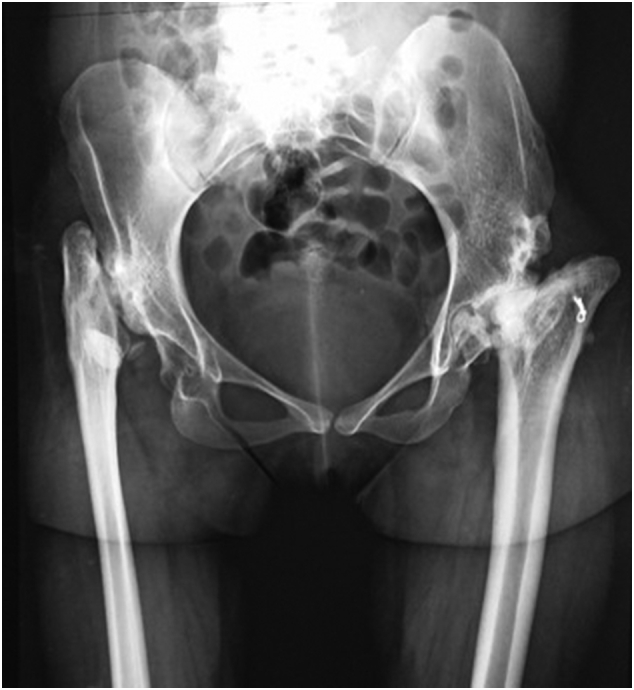
X-ray showing bilateral destruction of the hip joints which was more severe on the right side.

Examination revealed an apparent shortening of 1.5 cm on the right lower extremity which was supratrochanteric in origin. There was associated compensatory right sided pelvic tilt and scoliosis. All ranges of motion were restricted with only 10 and 12 degree of abduction in the left and right hip respectively. At presentation, the Harris Hip Scale (HHS) for the left and right hips was 53.4 and 46 respectively. X-rays showed bilateral destruction of the femoral head and neck and the acetabulum associated with subluxation. The subluxation was more severe on the right side with a high riding femoral head (Fig. 1). The Crowe classification of dislocation severity for the left and right hips were type III and IV respectively. The Choi classification for sequelae of septic arthritis of both hips was type IVB.

**Fig. 2:**
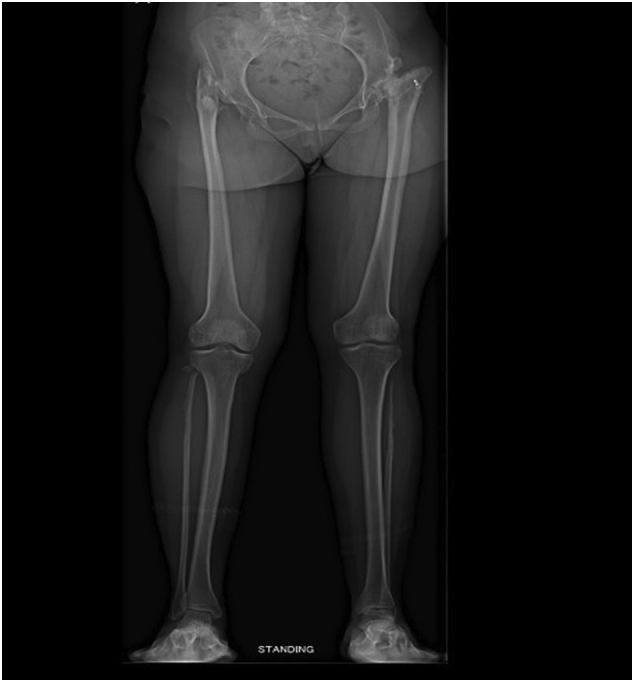
Scanogram showing supratrochanteric length discrepancy with a compensatory right sided pelvic tilt and scoliosis.

**Fig. 3:**
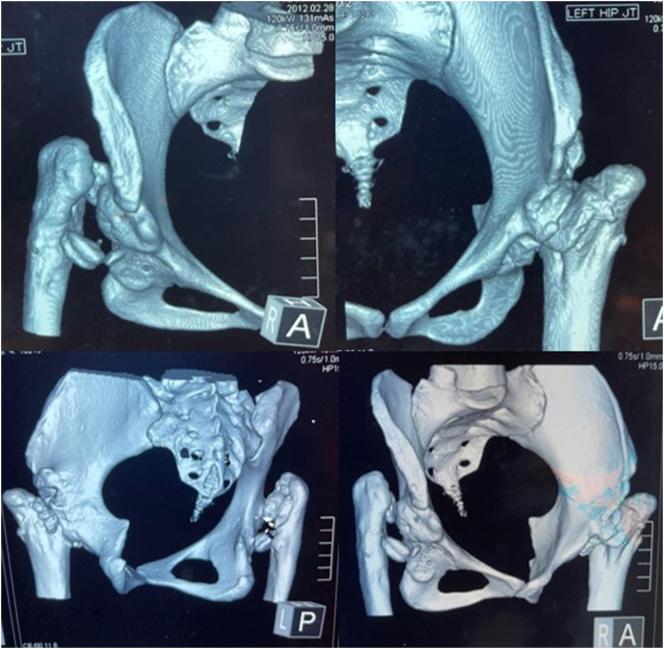
Computed tomography scan showing bilateral destruction of the femoral heads and neck and the acetabula. The left and right femoral canal sizes were 8 mm and 7 mm respectively.

Pre-operative planning was performed. A scanogram in standing position was done to assess the pelvic obliquity, scoliosis and length discrepancy of tibia, femur or both. The length discrepancy was contributed only by the supratrochanteric segment of the femur with a compensatory right sided pelvic tilt and scoliosis (Fig. 2). Computed tomography scan from hip to knee was performed to assess femoral canal size and femur and acetabular version (Fig. 3). There was bilateral destruction of the femoral heads and neck and the acetabula. The left and right femoral canal sizes were 8 mm and 7 mm respectively. The left total hip arthroplasty was performed first as it was more symptomatic. Furthermore, less problems were anticipated in the reduction of the head as it was Crow type III. The surgery was performed via standard posterior approach by a senior orthopedic surgeon in a tertiary care hospital. The sciatic nerve was preserved. The acetabular cup (48 mm) was placed in the true acetabulum. The S-ROM® modular hip system was used. The tissue fragments sent for culture were negative. The post-operative HHS was 95.7.

**Fig. 4:**
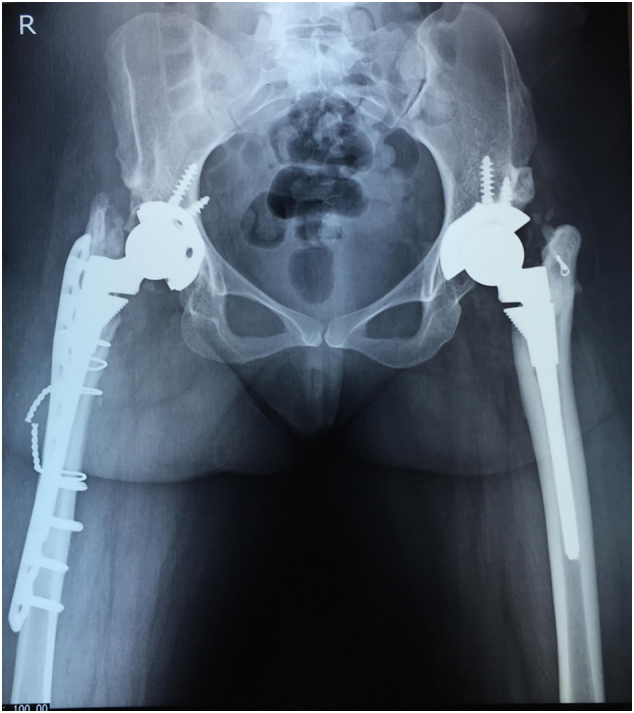
Post-operative X-ray after the second arthroplasty showing bilateral satisfactory reconstruction.

**Fig. 5:**
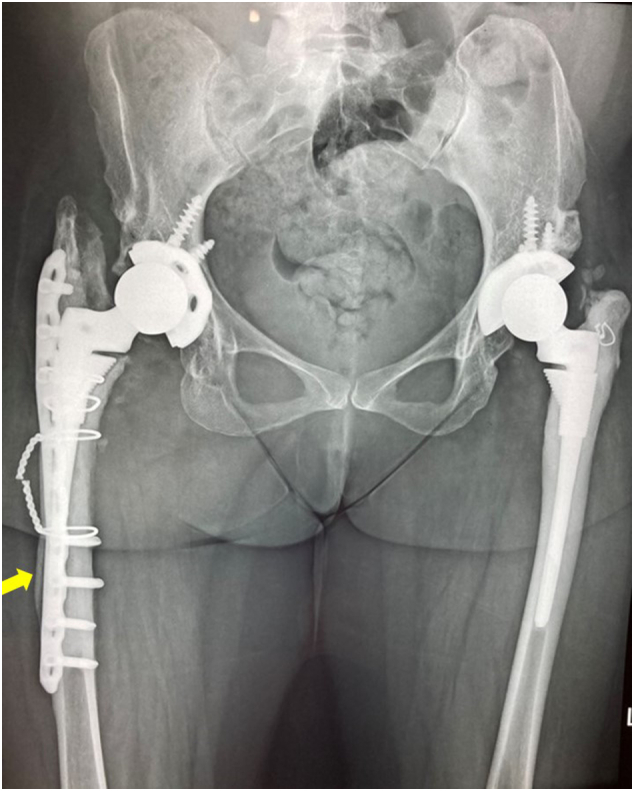
X-rays taken 5-years after the second arthroplasty. Note the healed fracture indicated by the arrow.

The high riding right hip was reconstructed 2 years later. A right total hip arthroplasty was also performed via standard posterior approach. The sciatic nerve was preserved. The acetabular cup (44 mm) was placed in the true acetabulum. A longitudinal split of the proximal femur was encountered during reaming. The femur was fixed with wire loops and a locking plate. The S-ROM® modular hip system was used. The tissue fragments sent for culture were negative. The post-operative HHS was 89.65. She underwent post-surgery rehabilitation with muscle strengthening and range of motion exercises. Fig. 4, Fig. 5 shows the post-op X-rays immediately after the second arthroplasty and 5 years later.

She sustained a fall 3 years after the last reconstruction but did not complain of pain in right hip or thigh. A healed peri-prosthetic fracture was incidentally noted in the subsequent X-rays taken 5 years after the last reconstruction. She is ambulatory and able to carry out her daily activities 5 years after the surgery.

## 3 Discussion

We described a patient who underwent a bilateral total hip arthroplasty for the severely damaged hip joints secondary to Tom Smith arthritis, with good long-term outcomes. We faced several challenges due to the late presentation of the patient with established deformity and the lack of suitable implants in our resource poor setting. Although the patient consulted several surgeons at her childhood and adolescence, surgical reconstruction was denied due to the severity of the disease and the lack of suitable implants in the government sector. Anatomical factors such as multiple previous surgeries, narrow medullary canals and possibility of breakage of thin femoral stems were also challenging. Furthermore, the patient was young and unmarried with long term expectations.

Detailed preoperative evaluation of the anatomy was paramount before surgical correction. Assessment of the limb-length discrepancy by means of scanogram and templating of the hip and proximal femur with computed tomography scan was useful. Planning the anatomical location of the centre of the relocated hip and predicting the use of shortening osteotomy were mandatory. Selection of appropriate implants and achievement of primary stability were essential. Acetabular cup should be ideally placed in the true acetabulum [9]. The next best option may be the placement of the acetabular cup within 2.0 cm distance above the inter teardrop line [9]. However, such placement may be associated with disadvantages such as incompetency of abductor mechanism, excessive joint reaction force and increased dislocation rate. Therefore, considering the young age and the long-term functional outcome, we opted to place both acetabular cups in the true acetabula. However, overzealous correction of the limb length discrepancy may lead to sciatic nerve traction. There is no consensus regarding the maximum correction that could be performed without injuring the sciatic nerve. Fortunately, our patient did not develop such complications.

The radiological classification was initially developed by Hunka et al. which was later modified by Choi et al. by reviewing 34 patients with infantile septic arthritis of the hip [6]. The prevalence of hip sequelae was higher in type II and type IV (type I: 15%, type II: 32%, type III: 15% and type IV: 38%) [6]. However, the relevance of such classification system in adults has not been studied due to the rarity of the disease. Therefore, we used the Crowe classification system and approached the case as a hip dysplasia [10]. Studies that described the surgical management of severe sequelae of infantile septic arthritis were exclusively performed in children [5], [6], [7]. Choi et al. reviewed 45 hips in 43 patients with severe complications of infantile septic arthritis. The average age at surgery was 5.9 years, ranging from 1.1–14.8 years of age [5]. Early realignment osteotomy or bone-grafting of the pseudoarthrosis in the proximal femur was performed for Choi type III disease. Trochanteric arthroplasty and Ilizarov's hip reconstruction osteotomy were performed for type IVA and IVB hips [5].

Wada et al. described the outcomes of 21 hips in 21 patients with severe complications due to Tom Smith arthritis. Similar to the previous study, the average age at surgery was only 4.2 years. Choi type IIIA and IV hips required femoral varus osteotomy with open reduction and pelvic osteotomy. However, the success rates were suboptimal [7]. These reports were only available in children and adolescents and the reports in adults with long-term severe sequelae of Tom Smith arthritis are limited. Furthermore, in our patient, bilateral hips had severe sequelae which were compatible with Choi Type IVB.

There are several learning points in this case report. This case report reveals that bilateral total hip arthroplasty is a feasible option to treat destructive hip disease caused by Tom Smith arthritis presenting in adulthood. The placement of acetabular cup should be decided pre-operatively weighing the pros and cons, and the patient's expectations. The surgery was performed via the posterior approach as it is the preferred approach of the surgeon. Furthermore, it allows good access and reduction is less cumbersome. Using the correct size prosthesis is important especially towards the femoral side to avoid inadvertent fracture due to the narrow medullary canal. Post-operative rehabilitation with fall prevention strategies are vital. Although our patient sustained a fall she was fortunate that the fracture was within the limits of the previous fixation and the plate was able to withstand the impact. Overall experience of the patient was satisfactory with good functional outcomes.

## 4 Conclusion

Reconstructive options for late sequelae should be individualized based on degree of involvement, hip stability, and patient expectations. Pre-operative assessment with scanogram and computed tomography is paramount in planning the reconstruction. The placement of acetabular cup should be decided pre-operatively weighing the pros and cons, and the patient's expectations. This case report reveals that bilateral total hip arthroplasty is a feasible option to manage the rare occurrence of severely damaged bilateral hip joints caused by Tom Smith arthritis presenting in adulthood.

## Abbreviations

TSA: Tom Smith arthritis
HHS: Harris Hip Scale

## Sources of funding

None declared.

## Ethics approval

Ethical approval is not required for publishing case reports in our institution.

## Consent

Written informed consent was obtained from the patient for publication of this case report and accompanying images. A copy of the written consent is available for review by the Editor-in-Chief of this journal on request.

## Author contributions

Author UJ and RS contributed to collection of information and writing of the manuscript. Authors UJ and RS contributed to writing and final approval of the manuscript. All authors read and approved the final version for publication.

## Research registration

Not applicable.

## Guarantor

Dr. Rukshan Sooriyarachchi, Senior Consultant Orthopedic Surgeon, Department of Orthopaedics and Trauma, National Hospital of Sri Lanka, Colombo, Sri Lanka.

## Declaration of competing interest

The authors declare that they have no competing interests.
